# The management of bone defect using long non-coding RNA as a potential biomarker for regulating the osteogenic differentiation process

**DOI:** 10.1007/s11033-021-07013-5

**Published:** 2022-01-01

**Authors:** Jia-lin Liu, Yan-shi Liu, Mei-jie Zheng, Hui-yu He

**Affiliations:** 1 Department of Prosthodontics, The First Affiliated Hospital of Xinjiang Medical University, Ürümqi, 830054 Xin Jiang Uygur Autonomous Region China; 2 Affiliated Stomatological Hospital of Xinjiang Medical University, Ürümqi, 830054 Xin Jiang Uygur Autonomous Region China; 3 Stomatology Research Institute of Xinjiang Uygur Autonomous Region, Ürümqi, 830054 Xin Jiang Uygur Autonomous Region China; 4 Department of Trauma and Microreconstructive Surgery, The First Affiliated Hospital of Xinjiang Medical University, Ürümqi, 830054 Xin Jiang Uygur Autonomous Region China

**Keywords:** Biomarker, Long non-coding RNA, Osteogenesis, Stem cell, Signaling pathway

## Abstract

Tissue engineered bone brings hope to the treatment of bone defects, and the osteogenic differentiation of stem cells is the key link. Inducing osteogenic differentiation of stem cells may be a potential approach to promote bone regeneration. In recent years, lncRNA has been studied in the field increasingly, which is believed can regulate cell cycle, proliferation, metastasis, differentiation and immunity, participating in a variety of physiology and pathology processes. At present, it has been confirmed that certain lncRNAs regulate the osteogenesis of stem cells and take part in mediating signaling pathways including Wnt/β-catenin, MAPK, TGF-β/BMP, and Notch pathways. Here, we provided an overview of lncRNA, reviewed its researches in the osteogenic differentiation of stem cells, emphasized the importance of lncRNA in bone regeneration, and focused on the roles of lncRNA in signaling pathways, in order to make adequate preparations for applying lncRNA to bone tissue Engineering, letting it regulate the osteogenic differentiation of stem cells for bone regeneration.

## Introduction

The reconstruction of maxillofacial bone defects provides a challenge in the medical field due to the inherent limitations. Tissue engineered bone brings a new turning point to resolve this intractable problem by combining biological scaffolds, seed cells and bioactive factors. On one hand, biological scaffolds have been improved continuously with antibacterial, mechanical and osteogenic potentials due to their advantageous morphology and physicochemical properties [1, 2]. On the other hand, it must be said that mesenchymal stem cells (MSCs), equipping with the potency of self-renewal and multidirectional differentiation, can be used as ideal seed cells for bone tissue engineering since they are usually regulated into osteoblasts for bone regeneration [3].

Genetically modified stem cells take part in the bone regeneration field, and long non-coding RNA (lncRNA) has become the research hotspot due to its importance in the regulation of MSCs differentiation, bone formation, and homeostasis [4]. Advantages including specific regulation of cell behavior, convenience of synthesis and operation, and non-inactivation. By regulating the activity of genes, lncRNA can affect the cell cycle, migration, invasion, proliferation, metastasis, immunity, and differentiation [5, 6], contributing to vital physiology and pathology processes. At present, it has been confirmed that lncRNAs can regulate the osteogenic differentiation of a variety of stem cells [7, 8]. Here, we hope to describe the current researches of lncRNAs about MSCs osteogenic differentiation, making adequate preparations for lncRNAs-regulated bone regeneration.

## The overview of long non-coding RNA

**Fig. 1:**
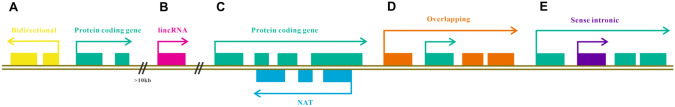
LncRNA classification diagram. **A** Bidirectional. It is transcribed in the opposite direction from their adjacent protein coding genes. **B** lincRNA. It is transcribed from the interval between two independent protein coding genes (within 1 kb). **C** NAT. It is transcribed from the antisense genomic strand of protein coding genes. **D** Overlapping. It is transcribed from the sense genomic strand of protein-coding genes. **E** Sense intronic. It is entirely transcribed from introns of protein-coding genes

LncRNA with a length of more than 200 nucleotides is considered as “transcription noise” without biological function in the initial stage on account of only participating in transcription and hardly translating proteins. Evidence accumulated over the past decade manifests that lncRNAs are mainly derived from mutations in encoding mRNA genes, chromosomal rearrangements, chromosomal duplication, and transposon insertion, are widely distributed in the nucleus and/or cytoplasm, has certain functions on transcriptional silencing, transcriptional activation, chromosome modification, and nuclear transport, these may be linked with their specific subcellular localizations [9]. Based on their subcellular localizations, the gene expression on diverse physiopathological process can be regulated by lncRNAs through interacting with DNA, RNA, and proteins. Back in 2013, Knauss et al. [10] classified lncRNA into five categories, namely Bidirectional, lincRNA, NAT, Overlapping and Sense intronic according to the location of lncRNA and protein coding gene (Fig. 1). This provides a perspective for a better understanding of the role played by lncRNA at different locations in various biological processes in cells. In addition, the expression of lncRNAs with cell specificity, tissue specificity and developmental stage specificity, suggesting that lncRNAs may be potential biomarkers for clinical targeting strategy.

**Fig. 2:**
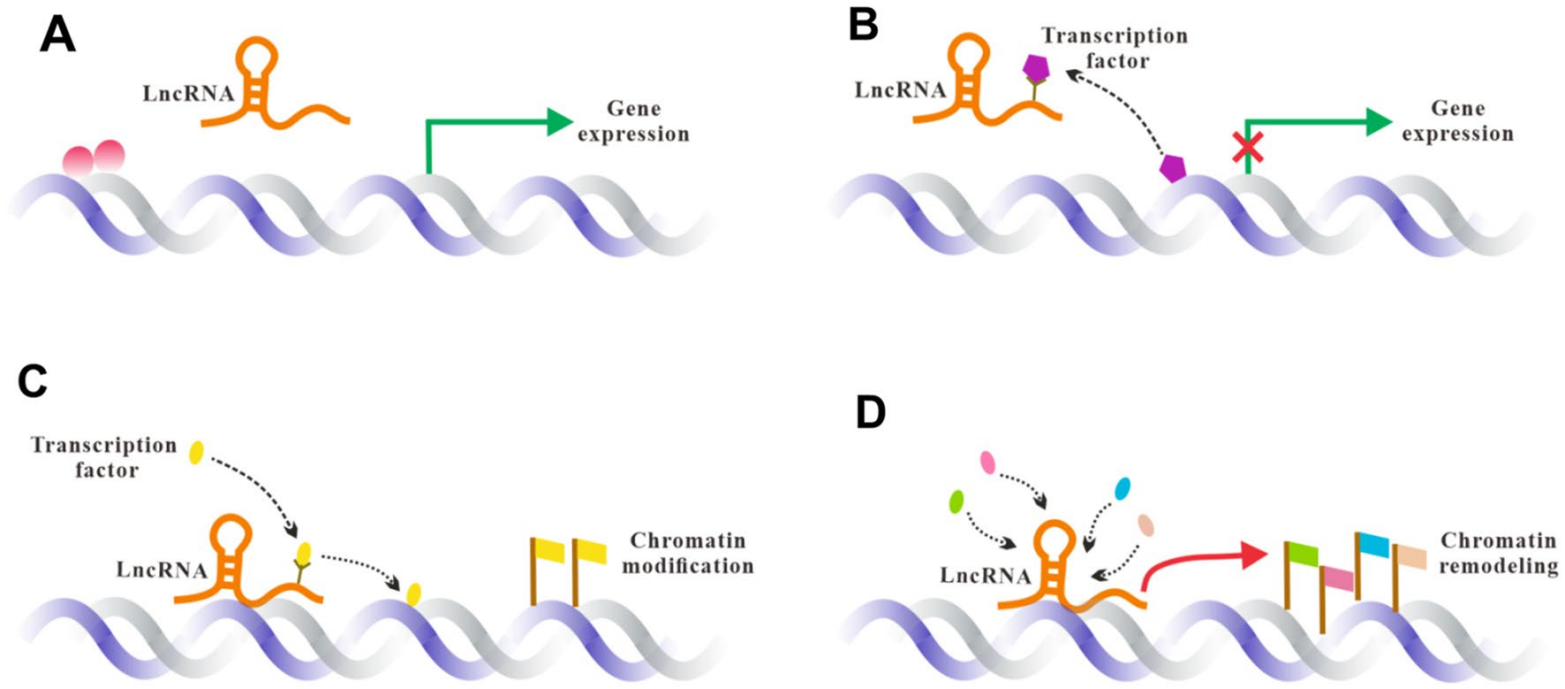
LncRNA regulatory modes. **A** Signal mode. LncRNA is specifically transcribed to participate in the conduction of special signal pathways, regulating the transcription of downstream genes. **B** Decoy mode. LncRNA blocks the effects of transcription factors and regulates the transcription of downstream genes by binding to transcription factors. **C** Guide mode. LncRNA combines with transcription factors, locating the transcription complex on specific DNA sequences, mediating the binding of transcription factors to specific DNA sequences. **D** Scaffold mode. Downstream effector molecules can be combined to the same LncRNA to achieve the information exchange and integration when multiple signaling pathways are activated at the same time

The regulatory modes of lncRNA are divided into four types: Signal, Decoy, Guide and Scaffold, which complement each other and play an important role in complex life activities (Fig. 2). Signal mode is common in a variety of stimulation and signal pathways, lncRNA is specifically transcribed to participate in the conduction of special signal pathways, regulating the transcription of downstream genes. In the Decoy mode, lncRNA blocks the effects of transcription factors and regulates the transcription of downstream genes by binding to transcription factors. In addition, lncRNA is rich in miRNA binding sites due to its homology with mRNA, which acts as a miRNA sponge in cells, alleviating the inhibitory influence of miRNA on its target gene and increasing target gene expression [11]. LncRNA combines with transcription factors, locating the transcription complex on specific DNA sequences in the Guide mode, thereby mediating the binding of transcription factors to specific DNA sequences [12]. The Scaffold mode always be seen when multiple signaling pathways are activated at the same time, downstream effector molecules can be combined to the same lncRNA to achieve the information exchange and integration, which is good for rapid response to external signals [7, 13].

From a functional point of view, lncRNA can play a cis- or trans-regulatory role in the pre-transcription, transcription, and post-transcription processes, affecting the biological processes of cells and the life activities of the body. LncRNA regulates histone acetylation, methylation and ubiquitination and DNA methylation at the pre-transcriptional level. LncRNA regulates the transcription level, interacts with enhancers, insulators, and transcription factors to influence the transcription of its neighboring genes, and its own transcription will also interfere the progress. At the post-transcriptional level, lncRNA participates in the regulation of pre-mRNA splicing, stabilizes ribonucleoprotein complexes and mRNA. Meanwhile, lncRNA is necessary to the miRNAs sponge procedure. Therefore, lncRNA may be used as a new regulatory molecule to influence various biological processes. At present, most researches on the function of lncRNA choose siRNA and shRNA interference, association analysis algorithm and CRISPR gene editing technology.

## The research status of lncRNAs related to osteogenic differentiation

**Fig. 3:**
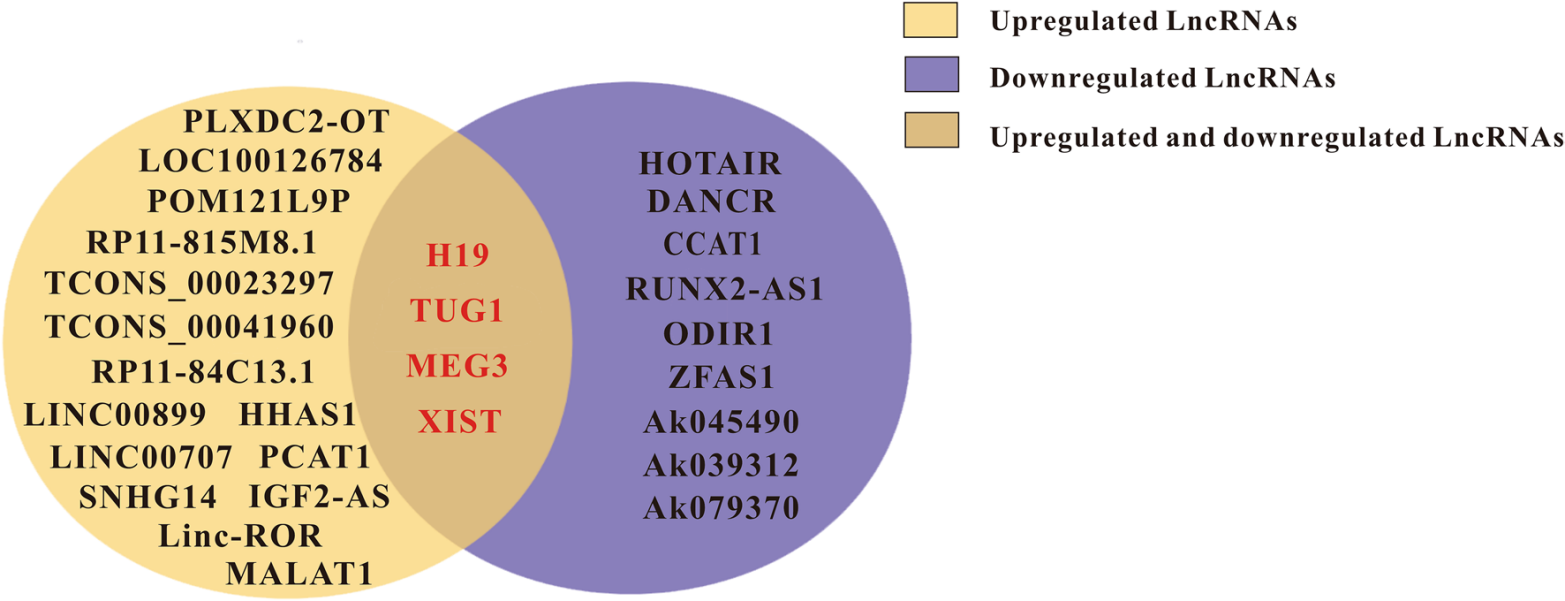
LncRNA upregulated and downregulated for osteogenic differentiation

With the continuous development of gene sequencing technology, the use of high-throughput sequencing combined with bioinformatics analysis has become a crucial scientific method for large-scale screening of lncRNAs related to osteogenic differentiation. Many scholars have compared the samples with and without osteogenic induction derived from diverse stem cells [14, 15] and then screened out differentially expressed lncRNAs, providing the scientific methodological basis and reliable data support for subsequent research. At present, we make the following summary of the research status of lncRNAs related to osteogenic differentiation (Fig. 3).

### LncRNA upregulated to promote osteogenic differentiation

#### LncRNA H19

LncRNA H19, one of the highly conserved members of cluster imprinting genes, is located on human chromosome 11, consistently expressed across multiple species. It is transcribed by RNA polymerase II, spliced, polyadenylated, and transported to the cytoplasm after capping, highly expressed in embryonic development. It has been confirmed that lncRNA H19 plays a critical role on various cancers, cardiovascular diseases, and osteogenic differentiation.

Recent research [13] has shown that the overexpression of lncRNA H19 can significantly enhance the osteogenic ability of hBMSCs. LncRNA H19 and its encoded miR-675 can block the phosphorylation of Smads3 downstream protein by inhibiting the expression of TGF-β, and prevent Smads3 from recruiting HDAC to target Runx2 [16]. When lncRNA and mRNA have the same miRNA response element (MRE), the competitive endogenous RNA (ceRNA) mechanism will be revealed. LncRNA can competitively adsorb miRNA and act as a “sponge” to reduce the inhibitory effect of miRNA on its target genes, increasing the target genes expression. The effect of lncRNA H19 were verified by regulating the miR-140-5p/SATB2 axis [17] and miR-149/SDF-1 axis [18] to promote osteogenesis. Studies on lncRNA H19 have demonstrated that its osteogenic effect is to activate the Wnt/β-catenin signaling pathway in combination with miR-141, miR-22 and miR-541-3p [7, 19]. To explore other function mechanism, Wu et al. [20] showed that lncRNA H19/miR-185-5p/IGF1 act as a positive regulatory axis in modulating matrix mineralization in osteoblasts. The balance between adipogenic differentiation and osteogenic differentiation of stem cells is particularly important for osteogenesis. The inhibitory effect of miR-188 on LCoR was reversed by binding to lncRNA H19, stabilizing the balance between adipogenic and osteogenic differentiation of mBMSCs [21]. In the process of searching osteoporosis therapeutic target, lncRNA H19 was revealed to increase hBMSCs proliferation and differentiation by regulating miR-19b-3p [22]. Besides, lncRNA H19 can also mediate the tension-induced osteogenesis in hBMSCs through combing with miR-138 to target PTK2 [23]. In summary, the above studies proved that lncRNA H19 could promote osteogenic differentiation, and ceRNA mechanism also made outstanding contributions.

#### LncRNA MALAT1

As one of the most well-known lncRNAs, MALAT1 is located on human chromosome 11q13, originally found in non-small cell lung cancer, and highly expressed in a variety of tumors [24]. It is considered as a biomarker of multiple tumors which can promote tumor cell proliferation, metastasis and invasion. At the same time, it is believed to be related to angiogenesis [25] and osteogenesis [26].

LncRNA MALAT1 up-regulated the expression of Smad4 by sponging miR-204, enhancing the osteogenic differentiation of hAVICs [27]. To explore differentially expressed lncRNAs of hBMSCs from patients with femoral head necrosis, high-throughput sequencing results showed that the downregulation of lncRNA MALAT1 may be related to the decrease of osteogenic differentiation [28]. Subsequently, lncRNA MALAT1 was found to promote osteogenesis by upregulating ATF4 through sponging miR-214, reversely verified the previous study [29]. Another two miRNAs that can bind to is miR-143 and miR-96, they have been proved to regulate the expression of the transcription factor osterix related to osteogenesis [26, 30]. In hADSCs, lncRNA MALAT1 can prompt Runx2-mediated osteogenic differentiation by targeting miR-30 [31]. Moreover, the overexpression of lncRNA MALAT1 can down-regulate miR-124 to promote the osteogenic ability of C3H10T1/2 cells derived from mouse embryos [32]. To improve the ossification of the posterior longitudinal ligament, it is also achieved by knocking down lncRNA MALAT1, releasing miR-1, and inhibiting the expression of CX43 gene [33]. In vivo, evidence displayed that lncRNA MALAT1 is low-expressed in osteoporotic rats. Inhibiting its expression may prevent osteogenesis by the activation of MAPK signaling pathway [34]. For osteoporotic mice, lncRNA MALAT1 was proved to enhance the osteoblast activity by mediating the miR-34c/SATB2 axis [35]. In general, lncRNA MALAT1 is a positive regulator of osteogenesis process.

#### LncRNA TUG1

LncRNA TUG1, located on chromosome 22q12, was initially identified as an essential gene for retinal development and photoreceptor formation in mouse. The imbalance of lncRNA TUG1 is link with the occurrence of tumors [36]. Also, it has a therapeutic effect on cardiovascular diseases. Its inhibition can reduce atherosclerosis and promote atherosclerotic vascular injury repairing [37].

For the aortic valve, lncRNA TUG1 can regulate Runx2 by sponging miR-204-5p to increase calcification [38]. In PDLSCs, the expression of lncRNA TUG1 is positively correlated with osteogenic differentiation [39, 40]. Further research demonstrated that lncRNA TUG1 accelerates the osteoblasts differentiation by sponging miR-545-3p and increasing CNR2 expression [41]. Teng, Z., et al. [42] verified lncRNA TUG1/miR-23b/Runx2 signaling prompt the osteogenic differentiation of BMSCs, it might provide a new insight for the diagnostic and therapeutic strategies for osteoporosis. Moreover, lncRNA TUG1 also down-regulated bFGF protein expression for the osteogenic capacity of TSPCs by promoting bFGF ubiquitination [43]. In contrast, Zhang, W et al. [44] proved that lncRNA TUG1 interacts with the 50–90aa region of Smad5 and prevents the nuclear translocation of p-Smad5, eliminating the osteogenic signal of hBMSCs after radiation. This seems contradictory, and may have relation to intervention factors or the type of cells. Generally speaking, lncRNA TUG1 has a positive role in regulating osteogenesis in certain cells, but this may be associated with cell types, because its cell specificity and different interventions may affect its specific role.

#### Other positive lncRNAs

Other lncRNAs, such as PLXDC2-OT, associated with the SIRT7/RBM6 protein complex to diminish its binding and deacetylation on OSX promoter and its inhibition to OSX, increasing osteogenic potential [45]. Usually, lncRNAs and miRNAs exert mutual interactions to control gene expression through ceRNA regulatory network. Thus, the role of miRNAs as regulators cannot be underestimated. Recent evidence has shown that lncRNAs LOC100126784 and POM121L9P were abundant in the cytoplasm, enhancing hBMSCs osteogenesis by improving SORBS1 expression via miR-503-5p combination [46]. The expression of lncRNA RP11-815M8.1 [47] was up-regulated after hBMSCs osteogenic induction in vitro, the conclusion is same as lncRNA SNHG14 [48]. When overexpressed lncRNA SNHG14, the crosstalk balance with miR-185-5p network will be broken, resulting in the increase of WISP2 expression for a better osteogenic effect. LncRNA IGF2-AS functioned as a sponge of miR-3126-5p to regulate KLK4 expression and positively modulated osteogenesis [11]. Previous studies also have shown that the stable expression of miR-138 or miR-145 decreases bone regeneration, but Linc-ROR can increase ZEB2 expression to enhance the process by acting as competitive endogenous RNAs for miR-138 and miR-145 [49]. Another study [50] clarified that lncRNA PCAT1 regulates the expression of miR-145-5p and its target TLR4 to improve hADSCs osteogenic differentiation. In addition, LINC00707 was extensively studied owing to its osteogenic potency by combining with miR-145 [51], miR-370-3p [52] or miR-103a-3p [53]. Notably, osteogenic differentiation, adipogenic differentiation, and osteogenesis–angiogenesis coupling of hBMSCs become new breakthrough points in the treatment for bone regeneration. LncRNA TCONS_00023297/miR-608/RUNX2/SHH signaling was proved a key regarding to the above biological functions [54]. Similarly, lncRNA TCONS_00041960 regulated Runx2 and GILZ by interacting with miR-204-5p and miR-125a-3p, respectively, enhancing the osteogenic effect and inhibiting its lipogenic effect of rBMSCs [55]. Furthermore, lncRNAs HHAS1 [56], RP11-84C13.1 [57], and LINC00899 [58] enhanced Runx2 level by downregulating miR-204-5p, miR-23b-3p, and miR-374a expression respectively to facilitate osteogenic differentiation. The aforementioned up-regulation of multiple lncRNAs can promote osteogenesis through the ceRNA mechanism, which illustrates its universality and importance.

### LncRNA upregulated to weaken osteogenic differentiation

#### LncRNA MEG3

LncRNA MEG3, is located on chromosome 14q32.3 with a length of about 1600 nucleotides. Previously, it was considered a possible therapeutic target for a variety of tumors. An increasing number of studies have shown it serves an essential part on osteogenesis. LncRNA MEG3 was highly expressed in osteoporosis patients and was positively correlated with the expression miR-133a-3p, which was accompanied by significant decline in SLC39A1 expression, inhibiting bone formation [59]. Results on in-vitro and in-vivo experiments indicated that down expression of lncRNA MEG3 could promote the osteoblast differentiation and fracture healing, it might be mediated by the Wnt/β-catenin signaling pathway [60, 61]. Additionally, lncRNA MEG3 silencing and miR-214 overexpression increased BMD, BV/TV, Tb.N, Tb.Th, trabecular bone area, collagen area and OPG expression [62], suggesting that it is a negative regulator. Different from aforesaid, some scholars [63] believed that knocking down lncRNA MEG3 would weaken the expression of osteogenic markers such as Runx2, Osterix and OCN, while overexpression would reverse the function [64]. Contradictory conclusions may have relationship with the disease state. The functions of lncRNA MEG may be different in certain states, indicating the complexity of its mechanism.

#### LncRNA XIST

LncRNA XIST, a momentous lncRNA concerning to the deactivation of chromosome X in mammals. In recent years, it has been attested to be associated with tumors development and progression by modulating cell proliferation, invasion, migration and apoptosis. Besides, it is crucial for bone regeneration. Chen, X et al. [65] indicated that overexpression of lncRNA XIST significantly inhibited osteoblast differentiation, as evidenced by the decrease of ALP, Bglap and Runx2. It may regulate axis miR-29b-3p/NNMT, miR-203-3p/ZFPM2, and miR-19a-3p/HOX5 to suppress the osteogenic function [66–68]. In contrast, the results of another two studies [69, 70] suggested that lncRNA XIST could be used as a positive regulator by targeting miR-9-5p/ALPL and miR-17-5p/AHNAK/BMP2 signaling.

#### Other negative lncRNAs

Here are other lncRNAs that weaken osteogenic differentiation after upregulation. In vitro experiment, lncRNA DANCR was reduced during the osteogenic induction, knocking down it can boost the function by regulating miR-1301-3p/PROX1 axis, revealing that it is a negative regulator [71]. LncRNA CCAT1 was found to prevent smurf2 degradation by bonding with miR-34a-5p, thereby suppressing the proliferation and differentiation of osteoblasts [72]. LncRNA RUNX2-AS1, from the antisense strand of Runx2, can form an RNA duplex with Runx2 pre-mRNA in the overlapping region, which transcriptionally inhibit Runx2 expression by reducing splicing efficiency, resulting in a reduction in the osteogenic potential of MSCs [73]. In hUC-MSCs, knockdown of lncRNA ODIR1 can promote osteogenic differentiation, while overexpression is the opposite effect [74]. From point of mechanism, lncRNA ODIR1 interacts with FBXO25 and promotes proteasome-dependent degradation of FBXO25 by recruiting CUL3. FBXO25 increases the monoubiquitination of H2BK120 and subsequently promotes the trimethylation of H3K4. Both H2BK120ub and H3K4me3 form a loose chromatin structure, which induces the transcription of osterix, and increases the expression of osteogenic genes such as OCN, OPN and ALP. Osteogenisis and adipogenisis are completely opposite concepts. Promoting osteogenic differentiation and inhibiting adipogenic differentiation are extremely important for bone formation. The down regulation of lncRNA ZFAS1 can not only facilitate osteoblasts differentiation, but also antagonize the positive effect on adipogenesis through a key regulator miR-499 [75]. In vivo, knocking down lncRNAs HOTAIR, AK045490, AK039312 or AK079370 also can partially alleviate osteoporosis [76–78]. In short, the above-mentioned down-regulation of multiple lncRNAs can motivate osteogenic differentiation. However, its specific effect may be different under various disease states, and the complexity of its mechanism is worthy of in-depth exploration.

## Signal pathways involving lncRNA-mediated osteogenesis

**Fig. 4:**
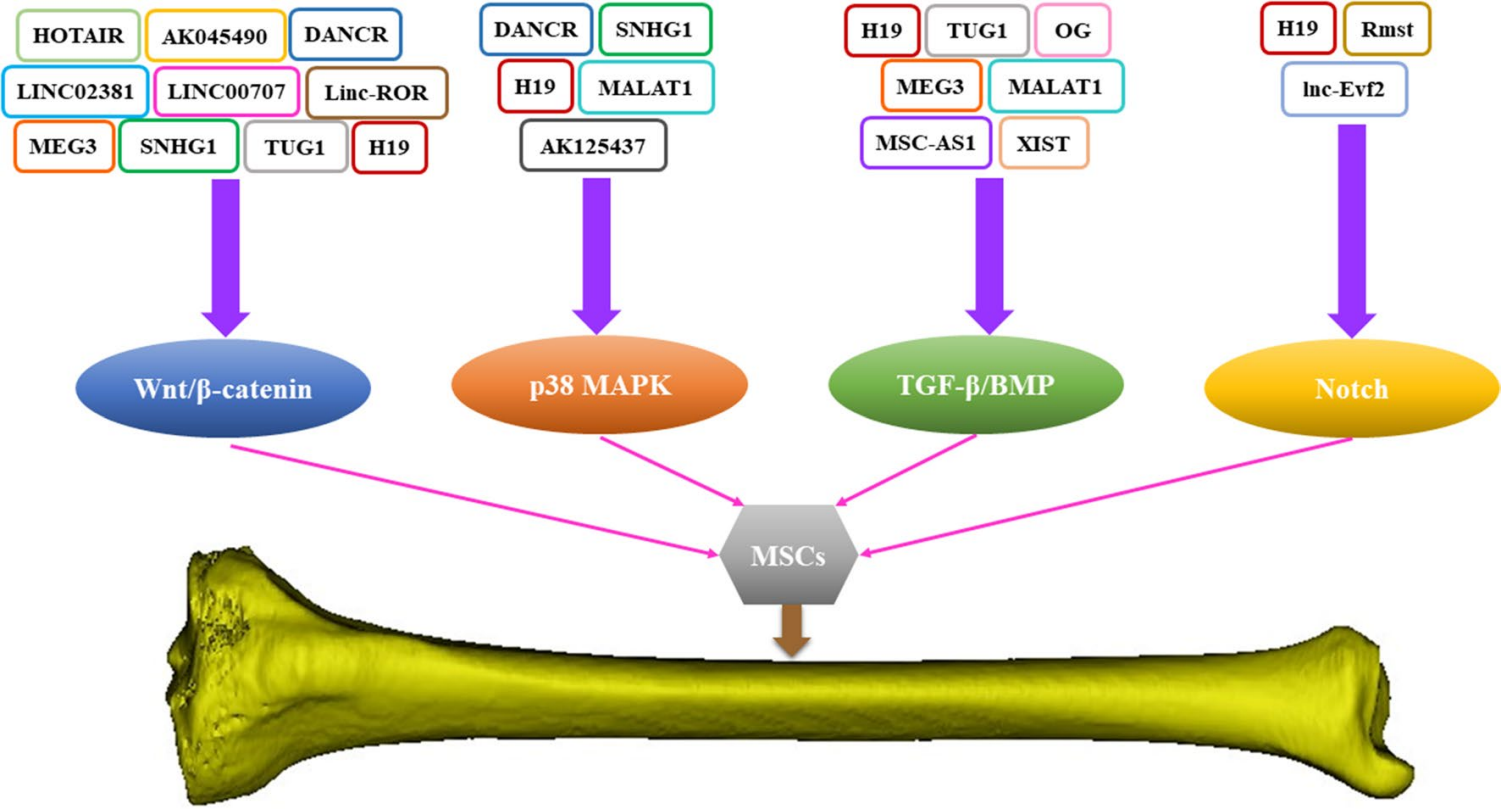
LncRNA regulates osteogenesis through four classical pathways

As is well-known, osteoblast differentiation is regulated by a variety of genes, involving multiple signaling pathways, such as Wnt/β-catenin pathway, MAPK pathway, TGF-β/BMP pathway, and Notch pathway, which play irreplaceable roles in many physiological and pathological processes. As recognized regulatory genes, lncRNAs widely participate in the mediation of diverse signaling pathways, may provide potential targets for bone regeneration (Fig. 4).

### Wnt/β-catenin signaling pathway

The Wnt/β-catenin signaling is a conventional pathway for bone homeostasis. Activating this pathway not only promotes osteogenic differentiation by opposing to adipogenic differentiation [79], but also influences BMPs signaling pathway in a positive feedback loop, facilitating osteoblast differentiation and bone formation [80]. It has been found that Wnt1, Wnt2, Wnt2b, Wnt3, Wnt3a, Wnt4, Wnt5a, Wnt5b, Wnt7b, and Wnt10b are closely related to osteogenesis. In terms of mechanism concerning to classic pathway, wnt ligands bind to Frizzled or LRP5/6 receptors to stimulate Dsh and inhibit the activation of compound Axin-APC-GSK3β, declining phosphorylation of β-catenin, which helps stabilize β-catenin expression. Next, β-catenin trans-localizes in the nucleus and binds to the DNA binding protein of T cell factor/lymph enhancer factor (TCF/LEF), regulating the osteogenic transcription factors Runx2 and Osterix expression. This signaling pathway is also negatively regulated by several antagonists, including Dkk1, SOST and SFRP.

Previous studies have proved that traditional lncRNAs such as H19, TUG1, Linc-ROR, MEG3, DANCR, and HOTAIR regulate the osteogenisis through Wnt/β-catenin pathway [7, 49, 61, 77, 81, 82]. Currently, three studies on LINC00707 showed that its target axis miR-145/LRP5, miR-370-3p/Wnt2b, and miR-103a-3p/Dkk1 are considered in the connection with Wnt/β-catenin signaling pathway for osteogenic differentiation [51–53]. Specific speaking, enforced expression of LINC00707 upregulates the level of LRP5 and wnt2b through binding miR-145 and miR-370-3p, respectively, positively act on Wnt/β-catenin signaling pathway. Meanwhile, reducing its expression and raising miR-103a-3p could cut down Dkk1 level which act as an antagonist, enhancing the motivation of this pathway. LINC02381, a sponge of miR-21, weakened osteogenic differentiation of hUC-MSCs through KLF12-mediated Wnt4 transcriptional repression [83], was related to the inactivation of Wnt/β-catenin signaling pathway. As one of antagonists referring to Wnt signaling pathway, SFRP1 could exert inhibitory function by competitively binding to the Frizzled receptor. LncRNA SNHG1 induced by SP1 combines with miR-181c-5p to increase SFRP1 activity, inhibiting Wnt signaling pathway mediated bone remodeling and angiogenesis [84]. Besides, lncRNA AK045490 inhibited the nuclear translocation of β-catenin protein to inactivate TCF1 and LEF1, reducing Runx2 expression in osteogenic differentiation [76]. Therefore, the Wnt/β-catenin signaling pathway has been proved a key in the osteogenesis process which may be regulated by lncRNAs directly or indirectly.

### MAPK signaling pathway

The MAPK signaling pathway is mainly involved in the transduction of a variety of extracellular stimuli, and can mediate cellular processes to make corresponding responses and adaptations, causing cell growth, differentiation and apoptosis. Traditional mitogen-activated protein kinases (MAPKs) include three subfamily members: (1) ERK1/2, ERK5; (2) JNK1/2/3; (3) p38. The p38 MAPK signal transduction is most closely link with osteogenesis and can be activated by the UV damage, oxidative stress, growth factors and cytokines. This pathway can mediate osteogenic differentiation, extracellular matrix deposition and mineralization after being activated by the BMPs, Wnts, and PTH effect, as evidenced by the usage of selective p38 inhibitors [85].

LncRNA SNHG1 not only regulated Wnt/β-catenin signaling, but also played a crucial role on p38 MAPK pathway. Overexpression of lncRNA SNHG1 enhanced the interaction between Nedd4 and p-p38, disrupted protein stability of p-p38, and promoted the ubiquitination. In addition, its down-regulation reversed the trend through reducing Nedd4 to elevate osteogenic level, while p-38 inhibitor abrogated the effects. In this way, lncRNA SNHG1 negatively regulated the p38 MAPK signaling pathway through Nedd4-mediated ubiquitination and suppresses the osteogenesis, both in vitro and in vivo [86]. Additionally, lncRNA DANCR was also related to p38 MAPK pathway when involved in the proliferation and osteogenic differentiation of hBMSCs [87]. In order to clarify the relationship between lncRNA DANCR and p38 MAPK pathway, lncRNA DANCR knockdown and overexpression vectors were transfected and treated with p38 inhibitors. The overexpression of lncRNA DANCR caused the inhibition of p38 MAPK pathway. Applying the inhibitor, the inhibitory effect of this pathway is reversed by knockdown of lncRNA DANCR, indicating that the up-regulation of lncRNA DANCR can negatively exert osteogenic effect by inhibiting the p38 MAPK signaling pathway. Except for the lncRNAs mentioned above, other lncRNAs such as H19, MALAT1, AK125437 [34, 88, 89] also have been verified to be related to this pathway. Together, it can be seen that the p38 MAPK signaling pathway has a significant influence on lncRNA-regulated osteogenesis.

### TGF-β/BMP signaling pathway

TGF-β/BMP can activate smad-dependent and -independent signal transduction pathways for bone regeneration. Bone morphogenetic proteins (BMPs) belong to the TGF-β superfamily and are a type of pleiotropic cytokines, which can be divided into 4 subfamilies based on similar sequence and function: (1) BMP-2 and -4; (2) BMP-5, -6, -7, -8a and -8b; (3) BMP-9 and BMP-10; (4) BMP-3, -3b, -11, -12, -13, -14, -15, and -16. Among them, BMP-2, -4, -5, -6, -7, -9 can improve bone formation [90]. Usually, BMP signaling molecules bind to and activate BMPR-II, phosphorylating BMPR-I and the C-terminal DNA-binding domain of BR-Smads, which binds to Co-Smads for heterogeneous oligomers in the nucleus to interact with transcription factors and jointly regulate the expression of target genes. In addition, BMPs directly or indirectly express Runx2 through BR-Smads, which physically binds to Runx2 to induce osteogenic differentiation.

There are many lncRNAs such as H19, MALAT1, TUG1, MEG3, and XIST [13, 27, 40, 63, 70] have been reported significant to TGF-β/BMP signaling pathway. Mechanically, lncRNA MEG3 located near the BMP-4 gene may dissociate SOX-2 from the BMP-4 promoter, thereby activating BMP-4 transcription for osteogenesis. Zhang et al. [91] verified lncRNA MSC-AS1 and BMP-2 were significantly upregulated in osteogenic induction, while miR-140-5p was downregulated. Further research confirmed that co-silence of lncRNA MSC-AS1 and miRNA-140-5p reverses the inhibitory effect of lncRNA MSC-AS1 knockdown on protein levels of p-Smad1/5/8, Runx2 and Osterix, suggesting that lncRNA MSC-AS1 could regulate osteogenesis through BMP-2/Smad pathway. In addition, hnRNPK can increase promoter histone acetylation to promote lncRNA-OG transcriptional activity. LncRNA OG regulated the expression of BMP family proteins (BMP-2, -4, -6) and remarkably affected Smad1/5/8 phosphorylation levels to promote osteogenic differentiation by interacting with hnRNPK [92]. Consequently, the prominent position of TGF-β/BMP signaling pathway in lncRNAs-mediated osteogenic differentiation of MSCs can be exhibited.

### Notch signaling pathway

Notch signaling pathway is connected with cell development, proliferation, apoptosis, differentiation and homeostasis of multicellular organisms. When cognate ligands on the surface of adjacent cells bind to the receptor, the extracellular and transmembrane parts of the Notch receptor are hydrolyzed by TACE and γ-secretase, respectively, causing the intracellular part of the Notch receptor (NICD) to detach from the cell membrane and move into the nucleus in which NICD interacts with RBPJ and MAML to convert transcriptional repressors into activators, activating gene expression of downstream HES family, HEY family, etc. The Notch downstream gene Hes-1 can interact with Runx2 and enhance its transcriptional activity, and then, MAML, an activator of the Notch pathway, has been demonstrated to activate Runx2 transcription in bone [93].

Currently, lnc-Evf2 was considered a novel potential clinical target to promote osteogenic differentiation through the Notch signaling [94]. In BMP-9-stimulated osteogenic differentiation of mMSCs, lncRNA H19 was increased and decreased at the early and late stage, respectively. However, whether knockdown or overexpression of lncRNA H19, BMP-9-induced osteogenesis was restrained [95]. The further experiment reversed the inhibitory effect after lncRNA H19 impact in BMP-9-induced osteogenesis by activating the Notch pathway, and confirmed the increased expression of miRNAs targeting Notch ligands and receptors by lncRNA H19, suggesting that lncRNA H19 may act as a mediator to miRNAs that target Notch pathway, and its conclusion is similar to LncRNA Rmst [96]. Overall, the Notch signaling pathway acts a regulatory function on osteogenic differentiation, but the effect of lncRNAs on MSCs owing to this pathway is complex.

### LncRNA-based therapy for bone tissue engineering

In recent years, considering that lncRNAs can mediate diverse signaling pathways and regulate multiple osteogenic genes expression, scholars began to focus on the application of lncRNAs acting on MSCs in the field of bone tissue engineering. Even though, the use of lncRNAs combined with scaffold has only been achieved in several publications, the facts still need to be mentioned. Other than lncRNA PWRN1-209 which can improve the bone formation of MSCs on Ti implants [97], LOC103691336 was also found to be upregulated in magnesium-based biodegradable implants, and bond with miR-138-5p in MSCs to change the inhibitory effect of miR-138-5p on BMP2 expression [98]. In a word, lncRNAs can be served as biomolecules acted on MSCs and combined with scaffolds for promoting osteogenesis.

## Conclusions

The use of high-throughput sequencing technology combined with bioinformatics analysis has become the mainstream modality for large-scale screening of lncRNAs associated with osteogenic differentiation. LncRNAs were originally discovered in cancer and later expanded in the fields of neurology, cardiovascular, metabolism, and bone regeneration, enriching multidisciplinary development. The critical and complex biological functions exhibited by lncRNAs deserve more explorations. Here, we describe the source, classification, mechanism, and biological function of lncRNAs, summarize the current researches of osteogenesis-related lncRNAs as well as the signaling pathways involved, hoping to discover more mechanisms of lncRNAs regulating osteogenic differentiation of MSCs.

In conclusion, lncRNAs can coordinate different kinds of molecules and signaling pathways to act regulatory effects during osteogenic differentiation of MSCs. Therefore, it is urgent to characterize their roles. With more in-depth studies on lncRNAs in the field of bone tissue engineering, lncRNAs may become potential therapeutic targets for bone defects. LncRNAs regulate the behavior of stem cells, guide stem cells to grow in, induce osteogenesis, and realize “regeneration” in a real sense, providing a new approach for bone defects. However, the specific mechanisms of lncRNAs involved in bone reconstruction from many aspects still need to be continuously improved, which will be the focus in our subsequent research.

## Data Availability

Not applicable.
